# Post-Transplant Cyclophosphamide versus Anti-Thymocyte Globulin in Patients with Hematological Malignancies Treated with Allogeneic Hematopoietic Stem Cell Transplantation from Haploidentical and Matched Unrelated Donors: A Real-Life Experience

**DOI:** 10.3390/hematolrep16020023

**Published:** 2024-04-17

**Authors:** Bianca Serio, Gabriella Storti, Matteo D’Addona, Lidia Santoro, Camilla Frieri, Danilo De Novellis, Luana Marano, Giovanna De Santis, Roberto Guariglia, Ilenia Manfra, Eleonora Urciuoli, Serena Luponio, Serena Marotta, Denise Morini, Michela Rizzo, Fausto Palmieri, Nicola Cantore, Valentina Giudice, Antonio Maria Risitano, Carmine Selleri

**Affiliations:** 1 Hematology Unit, University Hospital “San Giovanni di Dio e Ruggi d’Aragona”, 84131 Salerno, Italy; bianca.serio@sangiovannieruggi.it (B.S.);; 2 Hematology Unit, Hospital “S. Giuseppe Moscati”, 83100 Avellino, Italyamrisita@unina.it (A.M.R.); 3 Department of Medicine, Surgery, and Dentistry, University of Salerno, 84081 Baronissi, Italy

**Keywords:** graft versus host disease, prophylaxis, hematopoietic stem cell transplantation

## Abstract

**Background:** Post-transplant cyclophosphamide (PTCY) is widely used as graft versus host disease (GvHD) prophylaxis in allogeneic hematopoietic stem cell transplant (HSCT) recipients, with reported clinical benefits in patients who underwent transplant from a matched unrelated donor (MUD). However, real-life data on clinical efficacy and safety of PTCY in haploidentical and MUD transplantations are still poor. **Methods:** In our real-life retrospective observational study, we included a total of 40 consecutive adult patients who underwent haploidentical or MUD HSCT for various hematological malignancies and who received PTCY (*n* = 24) or ATG (*n* = 16) as GvHD prophylaxis at Hematology Units from hospitals of Salerno and Avellino, Italy, and clinical outcomes were compared. **Results:** We showed protective effects of PTCY against disease relapse with the relapse rate after transplantation of 16% versus 50% in the ATG arm (*p* = 0.02). All-cause mortality was lower (36% vs. 75%; *p* = 0.02) and the 2-year overall survival was slightly superior in patients administered PTCY (61% vs. 42%; *p* = 0.26). **Conclusions:** We support the use of PTCY, even in a real-life setting; however, the optimization of this protocol should be further investigated to better balance relapse prevention and GvHD prophylaxis.

## 1. Introduction

Allogenic hematopoietic stem cell transplantation (allo-HSCT) is a potentially curative therapeutic strategy for several benign and neoplastic hematological disorders; however, acute and chronic graft versus host disease (GvHD) is the principal post-transplant cause of death, leading to multi-organ failure [1]. This complication is driven by an immune attack against recipient tissue promoted by the donor’s lymphocytes; thus, HLA matching is the main risk factor of GvHD development and severity, as GvHD incidence in HLA-matched–related donor (MRD) HSCT is >30% [2]. Unfortunately, matched donors are available in only 30% of cases, and other types of donors are needed, such as matched unrelated (MUD) or haploidentical donors [3]. In these cases, GvHD incidence is high, and an effective prophylaxis is required and is still an unmet need [4]. Several approaches have been evaluated to inactivate alloreactive donor lymphocytes and to reduce acute and chronic GvHD incidence and severity, without increasing the risk of disease relapse [5].

Rabbit and horse polyclonal immunoglobulins directed against human T cell epitopes, the so-called anti-thymocyte globulins (ATGs), are currently used in combination with other immunosuppressors, such as cyclosporine A (CsA), methotrexate (MTX), sirolimus, tacrolimus, and mycophenolate mofetil (MMF) [6,7], for GvHD prophylaxis, showing a marked reduction in graft incidence and clinical benefits with decreased risk of relapse and non-relapse mortality (NRM) [8]. Indeed, ATG is considered a valid option for GvHD prevention in not-MRD allo-HSCT recipients; however, ATG induces prolonged immunosuppression with a significantly increased risk of bacterial and viral infections and/or viral reactivation, such as cytomegalovirus (CMV) [9]. For these reasons, other approaches have been investigated to improve transplant outcomes, including post-transplant high-dose cyclophosphamide (PTCY) on days +3 and +4, displaying reduced risks of graft rejection and valid GvHD prophylaxis due to the in vivo depletion of donor alloreactive lymphocytes [10].

Other clinical trials have described efficacy of PTCY for GvHD prophylaxis in patients with acute lymphoblastic leukemia (ALL) who underwent HSCT from MUD, while real-life data on clinical efficacy and safety of PTCY in haploidentical and MUD HSCT are still poor. In this real-life retrospective multi-center experience, we compared clinical outcomes, GvHD occurrence, risk of relapse, and safety of patients who received PTCY to those who had ATG as GvHD prophylaxis in not-HLA identical HSCT.

## 2. Materials and Methods

In our real-life retrospective observational study, we included a total of 40 consecutive adult patients who underwent haploidentical or MUD HSCT for various hematological malignancies and who received PTCY (*n* = 24) or ATG (*n* = 16) as GvHD prophylaxis at Hematology Units from hospitals of Salerno and Avellino, Italy, between 2009 and 2021. The median age was 51 and 45 years in PTCY and ATG groups, respectively, and patients received a diagnosis of acute myeloid leukemia in 63% and 75% of cases, ALL in 21% and 25%, and multiple myeloma (MM) in 12% and 0 of cases in PTCY and ATG arms, respectively (Table 1). All patients were in hematological complete remission at the transplant time. The GvHD grade was defined according to the modified Glucksberg or revised Seattle criteria for acute or chronic GvHD, respectively [11,12]. Engraftment was defined as previously reported [13], and intensity of conditioning regimens was chosen according to EBMT criteria and to intravenous busulfan dose reduction [14]. Patients in the PTCY cohort were treated with intravenous cyclophosphamide at a dose of 50 mg/kg on days +3 and +4, CSA, and MMF, while patients in the ATG group received a cumulative ATG dose of 7.5–10 mg/kg before stem cell infusion, MTX, CSA, or tacrolimus.

The primary endpoint was incidence and severity of acute and chronic GvHD. Secondary endpoints were the relapse incidence, relapse-free survival (RFS), overall survival (OS), graft failure rate, time to neutrophil and platelet engraftment. Data were collected in spreadsheets and analyzed using R software (v. 4.0.5; RStudio, Boston, MA, USA) and SPSS (v. 25; IBM, Armonk, NY, USA). Parametric and non-parametric tests were performed to compare continuous (*t*- and Mann–Whitney U test) and categorical variables (Chi square or Fisher’s exact test). Probabilities of RFS and OS were computed by the Kaplan–Meier method, and a log-rank test was used for comparisons. Univariate logistic regression models were employed to investigate effects and relative odds ratios of independent variables on GvHD or relapse incidence. To mitigate the impact of confounding factors, a multivariate regression analysis was performed, and Nagelkerke’s R-square test was used to assess model validity. A *p* value < 0.05 was considered statistically significant.

## 3. Results

Clinical and transplant features are summarized in Table 1; however, no statistically significant differences were observed in demographics between patients administered PTCY and ATG, except for stem cell source, as patients administered PTCY more frequently received bone marrow stem cells (54% vs. 25%; *p* = 0.05), and for donor type, as a son/daughter or brother/sister were the most represented in PTCY (54%) or ATG (72%) cohorts, respectively (*p* = 0.001). Time to allo-HSCT was similar between groups (11 vs. 9 months, PTCY vs. ATG; *p* = 0.23), and myeloablative regimens were less frequently used in patients administered PTCY, although this difference was not significant, likely because of the small number of subjects per group (71% vs. 94%, PTCY vs. ATG; *p* = 0.07). Patients administered PTCY predominantly received thiotepa–busulfan–fludarabine (92%) as the conditioning regimen, while subjects administered ATG mostly had busulfan–fludarabine (56% of cases) (*p* = 0.001). Two MUD HSCTs involved PTCY as GvHD prophylaxis. The donor lymphocyte infusion (DLI) rate after transplantation was similar between groups (12% vs. 13%, PTCY vs. ATG; *p* = 0.97). Other agents used for GvHD prophylaxis were CSA plus MMF (*n* = 24; 100%) in the PTCY cohort, and CSA plus MTX (*n* = 10; 63%) or CSA plus tacrolimus (*n* = 6; 37%) in the ATG group.

**Figure 1:**
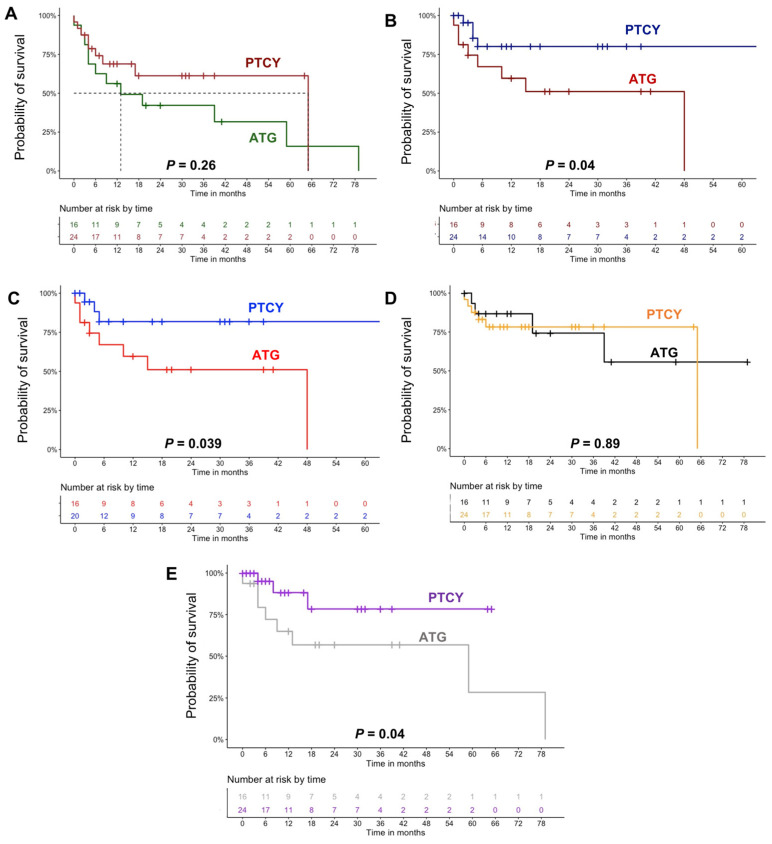
Clinical characteristics and outcomes. (**A**) Overall survival of patients who received post-transplant cyclophosphamide (PTCY) (dark red) or anti-thymocyte globulin (ATG) (dark green) for graft versus host disease (GvHD) prophylaxis in the entire cohort. Relapse-free survival of patients who received PTCY or ATG for GvHD prophylaxis in (**B**) the entire cohort (PTCY, dark blue; and ATG, dark red) and (**C**) only regarding patients with acute leukemia (PTCY, blue; and ATG, red). (**D**) Non-relapse mortality of patients who received PTCY (orange) or ATG (black) for GvHD prophylaxis in the entire cohort. (**E**) Relapse mortality of patients who received PTCY (violet) or ATG (grey) for GvHD prophylaxis in the entire cohort. Abbreviations. GvHD, graft versus host disease; PTCY, post-transplant cyclophosphamide; OR, odds ratio; CI, confidence interval; HSCs, hematopoietic stem cells; RIC, reduced-intensity conditioning.

Incidence of acute (46% vs. 37%; *p* = 0.72) and chronic (42% vs. 31%; *p* = 0.8) GvHD was not different between groups, with similar severity. The graft failure rate was lower in the PTCY group (8% vs. 19%; *p* = 0.32); however, time to engraftment was longer for neutrophils (20 vs. 16 days; *p* = 0.01) and platelets (29 vs. 16 days; *p* = 0.03) compared to the ATG group. The relapse rate after transplantation was significantly higher in the ATG arm (50% vs. 16%; *p* = 0.02), and 2-year relapse-free survival was shorter compared to PTCY (51% vs. 80%; *p* = 0.04). All-cause mortality was 36% vs. 75% (*p* = 0.02) while transplant-related mortality was 17% vs. 25% (*p* = 0.56) in PTCY and ATG cohorts, respectively, with 2-year overall survival (OS) being slightly superior in patients administered PTCY (61% vs. 42%; *p* = 0.26). Similar results were observed when comparing only patients with acute leukemias (Figure 1A–C). Next, non-relapse (NMR) and relapse mortality of patients who received PTCY or ATG as GvHD prophylaxis were compared between groups. No differences were observed for NRM between PTCY- and ATG-treated patients (*p* = 0.89), while ATG-prophylaxed subjects displayed a reduced relapse mortality compared to those treated with PTCY (*p* = 0.04) (Figure 1D,E).

Finally, by a univariate logistic regression analysis, a significant protective association against GvHD was observed in patients who received bone marrow stem cells (odds ratio [OR], 0.21; 95% confidence interval [CI], 0.05–0.86; *p* = 0.03), although it was not significant by a multivariate analysis (*p* = 0.28). Use of PTCY for GvHD prophylaxis demonstrated a strong anti-relapse effect both by univariate (OR, 0.2; 95%CI, 0.04–0.85; *p* = 0.03) and multivariate analyses (OR, 0.08; 95%CI, 0.01–0.89; *p* = 0.03) (Table 2). By uni- and multivariate linear regression analyses, the uses of PTCY (β, 4; 95%CI, 0.5–7.70; *p* = 0.03) and bone marrow stem cells (β, 4.1; 95%CI, 0.67–7.69; *p* = 0.02) were significantly associated with neutrophil engraftment time while not being associated with platelet engraftment time (Table 2 and Table 3).

## 4. Discussion

Use of PTCY for GvHD prophylaxis was proposed in 2008 for haploidentical HSCT, showing low incidence of acute GvHD without evidence of impaired transplant engraftment or immune reconstitution [2]. This evidence has confirmed by Solomon et al. in haploidentical HSCT recipients who received busulfan-based myeloablative regimens, peripheral blood as a stem cell source, and PTCY as GvHD prophylaxis [15], and by Bhamidipati et al. in non-myeloablative conditioning regimens [16], with cumulative incidences of acute and chronic GvHD lower than 50% (all grades) and 1-year OS greater than 60%. Because of these encouraging results in haploidentical HSCT, PTCY has also been employed in HSCT with MRD, MUD, and mismatched unrelated donors. Currently, PTCY is used in combination with other immunosuppressive agents, such as cyclosporine A and mofetil mycophenolate, for GvHD prophylaxis in haploidentical and MUD HSCT [17,18,19,20,21,22]. In patients with ALL treated with MUD HSCT, cumulative incidence of acute and chronic GVHD is similar between PTCY- and ATG-prophylaxed patients, as also confirmed in our study. Moreover, GvHD occurrence after PTCY was not different than that reported in the ATG group, regardless of underlying hematological malignancies—not only ALL—and type of HSCT donor, as the majority of our patients received HSCT from a haploidentical donor. However, Giebel et al. have described a reduced risk of extensive chronic GvHD in ATG recipients by a multivariate analysis (hazard ratio, 0.54; 95%CI, 0.3–0.98; *p* = 0.04) [1], not observed in our cohort. Although GvHD incidence was comparable between groups, we observed a significant protection from hematological relapse in the PTCY arm, likely because of a prompter T cell compartment and anti-tumor immune surveillance reconstitution [23]. Indeed, cyclophosphamide specifically removes regulatory T cells, a lymphocyte subpopulation responsible for immune tolerance, resulting in an enhanced anti-tumor cytotoxic activity [24,25]. Moreover, a faster engraftment is related to a quicker and better immunological reconstitution, and thus a reduced infectious complication rate in transplant recipients [26].

Giebel et al. have reported a lower cumulative incidence of relapse at 2 years (18% vs. 25%; *p* = 0.046), of leukemia-free survival rates (71% vs. 59%; *p* = 0.01), and of OS in the PTCY group (82% vs. 74%; *p* = 0.08) compared to ATG [1]. In our real-life study including different types of hematological malignancies, a significant protection from relapse was confirmed in PTCY-treated patients. In addition, when we restricted our analysis only to patients with acute leukemia, differences in the relapse rate and RFS remained unchanged, suggesting that underlying hematological conditions or use of reduced-intensity regimens in PTCY groups did not influence clinical outcomes. Finally, time to neutrophil and platelet engraftment was slightly longer in PTCY recipients compared to ATG-treated patients, even though incidence of graft failure was significantly higher in the ATG group. Similar results have been previously reported in mismatched MUD, MRD, and haploidentical donor HSCT [27,28]. PTCY as GvHD prophylaxis can induce cardiotoxicity as cardiomyopathy in 9–22% of cases, especially in the elderly with a comorbidity index score ≥4 [29,30,31]. In our study, we did not observe PTCY-induced cardiotoxicity, likely because of a younger age in our fit patients (mean age, 51 years old).

Our study has some limitations: (i) the retrospective nature of this work; (ii) a small sample size with some population heterogeneity, such as hematological disease type, stem cell source, donor type, conditioning regimen, and GvHD prophylaxis. Of note, GvHD prophylaxis regimens are frequently heterogeneous, especially outside clinical trials and in real-life retrospective observational studies [32,33,34], and conditioning regimens are chosen based on international guidelines, disease type, clinical decisions, and local institution guidelines. Nonetheless, the logistic regression analysis did not show a significant impact of these variables on GvHD incidence or disease relapse.

## 5. Conclusions

In conclusion, we confirmed that PTCY is more effective than ATG in relapse protection, regardless of underlying hematological malignancies and types of HSCT donor, even in a real-life setting; however, the optimization of this protocol should be further investigated to better balance relapse prevention and GvHD prophylaxis.

## Figures and Tables

**Table 1:** Patients’ characteristics.

Characteristics	PTCY Cohort	ATG Cohort	*p* Value
	*n* = 24	*n*= 16	
Median age, years (range)	51 (20–71)	45 (21–61)	0.39
Gender, *n* (%)			0.25
Male	11 (46)	5 (31)	
Female	13 (54)	11 (69)	
Hematologic malignancy, *n* (%)			0.63
AML	15 (63)	12 (75)	
ALL	5 (21)	4 (25)	
MM	3 (12)	-	
Other	1 (4)	-	
Stem cell source, *n* (%)			0.05
Peripheral blood	11 (46)	12 (75)	
Bone marrow	13 (54)	4 (25)	
Time to transplant, months, median (range)	11 (1–35)	9 (1–44)	0.23
Stem cells infused, ×10^6^/kg, median (range)	3.70 (0.60–7.90)	4.78 (1–8.29)	0.05
Type of donor, *n* (%)			0.001
Sister/brother	7 (30)	13 (72)	
Mother/father	2 (8)	2 (12)	
Son/daughter	13 (54)	1 (6)	
MUD	2 (8)	-	
CMV serostatus, *n* (%)			0.5
R+/D+	17 (71)	12 (75)	
R−/D+	3 (13)	2 (12)	
R+/D−	2 (8)	1 (6)	
R−/D−	2 (8)	1 (6)	
Conditioning regimen, *n* (%)			0.001
TBF	22 (92)	5 (31)	
BU-FLU	1 (4)	9 (56)	
FLU-MEL	1 (4)	-	
R-TBF	-	2 (13)	
Type of conditioning, *n* (%)			0.07
Myeloablative	17 (71)	15 (94)	
Reduced intensity	7 (29)	1 (6)	
Immunosuppressive drugs, *n* (%)			0.001
CSA + MTX	-	10 (63)	
CSA + tacrolimus	-	6 (37)	
CSA + MMF	24 (100)	-	
Engraftment failure, *n* (%)	2 (8)	3 (19)	0.32
Neutrophil engraftment, days, median (range)	20 (11–36)	16 (10–25)	0.01
Platelet engraftment, days, median (range)	29 (11–184)	16 (10–37)	0.03
Acute GvHD, sites, *n* (%)	11 (46)	6 (37)	0.72
Skin	8 (32)	4 (25)	
Intestine	6 (24)	1 (6)	
Liver	4 (16)	2 (13)	
Acute GvHD grading, *n* (%)			0.8
Grade I–II	10 (40)	5 (31)	
Grade III–IV	1 (4)	1 (6)	
Chronic GvHD, sites, *n* (%)	10 (42)	5 (31)	0.8
Skin	7 (28)	5 (31)	
Intestine	2 (8)	2 (13)	
Liver	4 (16)	1 (6)	
Lung	1 (4)	1 (6)	
Eyes	1 (4)	-	
Chronic GvHD grading, *n* (%)			0.7
Mild	6 (24)	3 (19)	
Severe	4 (16)	2 (13)	
Donor lymphocyte infusion, *n* (%)	3 (12)	2 (13)	0.97
Hematological relapse, *n* (%)	4 (16)	8 (50)	0.02
All-cause deaths, *n* (%)	9 (36)	12 (75)	0.02
Causes of death, *n* (%)			0.03
Relapse	3 (13)	8 (50)	
GvHD	3 (13)	1(6)	
Infections	3 (13)	1(6)	
Others	-	2 (12)	
Transplant-related mortality, *n* (%)	4 (17)	4 (25)	0.56
2-year relapse-free survival	80%	51%	0.04
2-year overall survival	61%	42%	0.26

**Abbreviations.** PTCY, post-transplant cyclophosphamide; ATG, anti-thymocyte globulin; AML, acute myeloid leukemia; ALL, acute lymphoid leukemia; MM, multiple myeloma; MUD, mismatched unrelated donor; D, donor; R, recipient; TBF, thiotepa–busulfan–fludarabine; BU-FLU, busulfan–fludarabine; FLU-MEL, fludarabine–melphalan; R-TBF, total body irradiation + TBF; CSA, ciclosporin A; MTX, methotrexate; MMF, mycophenolate mofetil; GvHD, graft versus host disease.

**Table 2:** Univariate logistic regression analysis.

Dependent Variable = GvHD Occurrence	OR	95%CI	*p* Value
PTCY [yes]	1.27	0.34–4.69	0.72
Gender [female]	1.52	0.41–5.61	0.52
Age, years	0.96	0.91–1.01	0.12
Bone marrow HSCs	0.21	0.05–0.86	0.03
Number of infused HSCs	1.36	0.95–1.96	0.09
Donor type [sister/brother]	1.71	0.47–6.16	0.40
RIC	1.37	0.27–6.67	0.69
Conditioning with TBF	0.9	0.5–2.45	0.8
**Dependent Variable = Relapse**	**OR**	**95%CI**	***p* Value**
PTCY [yes]	0.2	0.04–0.85	0.03
Gender [female]	0.9	0.22–3.58	0.88
Age, years	0.98	0.93–1.03	0.57
Bone marrow HSCs	0.57	0.14–2.36	0.44
Number of infused HSCs	1.21	0.86–1.70	0.27
Donor type [sister/brother]	2.6	0.64–10.9	0.17
RIC	1.34	0.23–7.97	0.73
Conditioning with TBF	0.7	0.4–2	0.5
**Dependent Variable = Neutrophil Engraftment**	**β**	**95%CI**	***p* Value**
Gender [female]	−0.3	−4.54/3.82	0.86
Age, years	−0.1	−0.24/0.04	0.17
PTCY [yes]	5	1.23/8.81	0.01
Bone marrow HSCs	5.23	1.59/8.88	0.01
Number of infused HSCs	−0.8	−1.8/0.15	0.09
Donor type [sister/brother]	−1	−5.1/3.02	0.61
RIC	0.24	−4.73/5.22	0.1
**Dependent Variable = Platelet Engraftment**	**β**	**95%CI**	***p* Value**
Gender [female]	3.7	−17.9/25.3	0.72
Age, years	0.32	−0.44/1.09	0.4
PTCY [yes]	17	0.5/35	0.04
Bone marrow HSCs	16.1	−3.7/34	0.1
Number of infused HSCs	−5.1	−10.3/−0.01	0.09
Donor type [sister/brother]	−0.2	−4.1/2.04	0.57
RIC	3.67	−24.4/31.8	0.89

**Abbreviations.** OR, odds ratio; CI, confidence interval; PTCY, post-transplant cyclophosphamide; HSCs, hematopoietic stem cells; RIC, reduced-intensity conditioning; TBF, thiotepa–busulfan–fludarabine.

**Table 3:** Multivariate logistic regression analysis.

Dependent Variable = GvHD Occurrence	OR	95%CI	*p* Value
Age, years	0.95	0.62–1.91	0.16
Bone marrow HSCs	0.29	0.03–2.75	0.28
Number of infused HSCs	1.09	0.62–1.91	0.74
**Dependent Variable = Relapse**	**OR**	**95%CI**	***p* Value**
PTCY [yes]	0.08	0.01–0.89	0.03
Age, years	0.98	0.92–1.05	0.72
Number of infused HSCs	1.1	0.72–1.66	0.65
Donor type [sister/brother]	2.03	0.59–6.98	0.25
**Dependent Variable = Neutrophil Engraftment**	**β**	**95%CI**	***p* Value**
Age, years	−0.1	−0.2/0.01	0.07
PTCY [yes]	4	0.5/7.70	0.03
Bone marrow HSCs	4.1	0.67–7.69	0.02
**Dependent Variable = Platelet Engraftment**	**β**	**95%CI**	***p* Value**
Age, years	0.26	−0.47/1.01	0.47
PTCY [yes]	4	−5.65/36.8	0.14
Bone marrow HSCs	11.9	−9.06/32	0.25

**Abbreviations.** OR, odds ratio; CI, confidence interval; PTCY, post-transplant cyclophosphamide; HSCs, hematopoietic stem cells.

## Data Availability

Data are available upon request to the authors.
